# Expansion of Intestinal Secretory Cell Population Induced by *Listeria monocytogenes* Infection: Accompanied With the Inhibition of NOTCH Pathway

**DOI:** 10.3389/fcimb.2022.793335

**Published:** 2022-03-25

**Authors:** Cong Zhou, Yuanyuan Zhang, Anthony Bassey, Jie Huang, Yafang Zou, Keping Ye

**Affiliations:** National Centre of Meat Quality and Safety Control, Jiangsu Collaborative Innovation Center of Meat Production and Processing, Quality and Safety Control, College of Food Science and Technology, Nanjing Agricultural University, Nanjing, China

**Keywords:** *Listeria monocytogenes*, intestine, goblet cell, Paneth cell, notch pathway

## Abstract

*Listeria monocytogenes*, as a model organism, is a causative agent of enteric pathogen that causes systemic infection. However, the interaction of *L. monocytogenes* and small intestinal epithelium has not been fully elucidated yet. In this study, mice and intestinal organoids were chosen as the models to investigate the influence of *L. monocytogenes* infection on the intestinal secretory cells and its differentiation-related pathways. Results confirmed the phenomenon of intestinal damage that *L. monocytogenes* infection could lead to villi damage in mice, which was accompanied by the increase of TNF-α production in jejunum as well as lipopolysaccharide (LPS) secretion in serum. Moreover, it was demonstrated that *L. monocytogenes* infection increased the number of goblet and Paneth cells in mice and intestinal organoids and upregulated the expression of *Muc2* and *Lyz*. Furthermore, *L. monocytogenes* decreased the relative expression of Notch pathway-related genes (*Jag1*, *Dll4*, *Notch1*, and *Hes1*) while upregulating the relative expression of *Math1* gene in mice and intestinal organoids. This indicated that *L. monocytogenes* infection caused the inhibition of Notch pathway, which may be the reason for the increased number of goblet and Paneth cells in the intestine. Collectively, these results are expected to provide more information on the mechanism of *L. monocytogene*s infection in the intestine.

## 1 Introduction

*Listeria monocytogenes* is a major foodborne pathogen that causes listeriosis and has a high mortality rate ranging from 20 to 30% (de Noordhout et al., 2014). Its severity is attributed to the fact that it can cross the intestinal, placental, and blood-brain barriers in immunocompromised individuals, inducing gastroenteritis, abortions, and meningitis (Doganay, 2003).

As the first defense barrier, the intestine plays a vital role in *L. monocytogenes* defense (Gahan and Hill, 2014; Becattini and Pamer, 2018). Under normal conditions, mucosal epithelial cells, mucus-secreting cells, and immune cells form a protective barrier against invading pathogens (Ruch and Engel, 2017). Related studies mainly focused on the protective effects of immune cells during *L. monocytogenes* infection (Cho et al., 2017; Wilharm et al., 2021). However, the intestinal epithelial cells compose numerous cell types that help maintain intestinal homeostasis and defend against enteric pathogen invasion. Specifically, goblet cells can produce mucin glycoproteins and form mucus, and Paneth cells, at the bottom of intestinal crypts, can secret antimicrobial peptides (AMPs). It had been found that *L. monocytogenes* could damage intestinal homeostasis and affect the differentiation of intestinal epithelial cells. A study on intestinal infection by *L. monocytogenes* demonstrated a significant effect on the differentiation of epithelial cells by increasing the number of goblet cells in mice (Pian et al., 2020). In addition, it was reported that the counts of Paneth cells in organoids decreased after *L. monocytogenes* infection for 1 h but increased after infection for 18 h through the regulation of Wnt signaling pathway (Huang et al., 2021). Therefore, these studies indicated that the differentiation of epithelial cells, especially the secretory cells, could be affected by bacteria invasion *in vivo* or *in vitro*.

In literature, the proliferation and differentiation of intestinal epithelial cells were confirmed to be regulated by a variety of signaling pathways, such as Wnt, EGF, BMP, and Notch signaling pathways (Date and Sato, 2015). Among them, the Notch signaling pathway played a crucial role in regulating the differentiation of intestinal secretory cells, such as goblet and Paneth cells. Recently, some studies have demonstrated that mice exposed to Cadmium or *Salmonella* infection led to a loss of goblet cells through the activation of Notch-signaling pathway (Wu et al., 2018; Xie et al., 2020). Conversely, blockade of the Notch pathway using γ-secretase inhibitors led to the conversion of all intestinal epithelial cells into goblet cells (Wong et al., 2004; van der Flier and Clevers, 2009). These studies indicated that the Notch signaling pathway regulated the differentiation of secretory cells under physiological and pathological conditions. However, researches on the influence of intestinal infection caused by *L. monocytogenes* on the Notch pathway are insufficient.

Animals and cells models are often used to investigate the infection mechanism of the enteric pathogens in the intestine. However, most traditional intestinal cell models have immortalized 2-D cell lines, such as Caco-2 cells containing a single cell type, which cannot reproduce some characteristics of natural infection (Wilson et al., 2015). Recently, intestinal organoids were served as a more effective model to study pathogen-host interactions (Hill and Spence, 2017). In contrast to traditional cell lines, intestinal organoids occupied 3-dimensional space. They formed complex microenvironments that facilitated differentiation and persistence of epithelial subtypes and the formation of villus-like structures, comprised of Paneth cells, Goblet cells, enterocytes, enteroendocrine cells, and stem cells (Watson et al., 2014; Finkbeiner et al., 2015). In addition, it was reported that intestinal organoids were used to visualize the invasiveness of *Salmonella* and the morphologic changes of the organoids (Zhang et al., 2014). And intestinal organoids also were used to model the infection of *L. monocytogenes* (Huang et al., 2021), and pathogenic *Escherichia coli* strains (Vandussen et al., 2014; Rajan et al., 2018), which provided important insights into the pathogenesis of the intestine.

Therefore, in this study, mice and intestinal organoids were used to establish an invasion model of *L. monocytogenes* to explore its influence on the differentiation of secretory cells and differentiation-related Notch pathway, which will provide more information on the mechanism of *L. monocytogenes* infection in the intestine.

## 2 Materials and Methods

### 2.1 Bacterial Strain Culture

The *L. monocytogenes* 10403s strain used in this study was supplied by Prof. Weihuan Fang (Zhejiang University). *L. monocytogenes* 10403s was grown in brain heart infusion (BHI) broth supplemented with 5 μg/ml erythromycin for 16 h at 37°C with shaking (180 rpm).

### 2.2 Animals and Intestinal Organoids

#### 2.2.1 Animals

Twenty-four C57BL/6 mice (4 weeks old, specific-pathogen-free (SPF) female) were purchased from the Animal Research Centre of Yang Zhou University. All animals were randomly divided into two groups and orally administrated sterile PBS (control group, CK, n=6) and *L. monocytogenes* 10403s (10^9^ CFU/ml, LM, n=18). The mice were sacrificed on day 4, and tissue samples were collected for further analysis. All animal studies were approved by the Nanjing Agriculture University Committee on Animal Resources Committee and the National Institutes of Health guidelines for the performance of animal experiments.

#### 2.2.2 Intestinal Organoids

##### 2.2.2.1 Isolation and Culture of Intestinal Organoids

Intestinal organoids were isolated from the small intestine of 4-week-old SPF C57/BL6 mice. The intestine samples were cleaned with phosphate-buffer saline (PBS) and cut into small pieces. After that, Gentle Cell Dissociation Reagent (Stem Cell, Canada) was added, and the mixture was digested at 20°C for 15 min. After incubation, crypts were filtered through a 70-μm sterile cell strainer and centrifuged at 300 g for 5 min at 4°C. The cells were resuspended by Matrigel (Corning, USA) and IntestiCult™ OGM Mouse Basal Medium (Stem cell, Canada) and then plated in 24-well plates. The plates were polymerized at 37°C for 20 min before the addition of culture medium. The medium was changed every 2-3 days.

##### 2.2.2.2 L. Monocytogenes Infection of Organoid Cells

The methods of organoids infection were referenced from Huang et al. (Huang et al., 2021) with some modifications. Firstly, *L. monocytogenes* culture was centrifuged at 5000 rpm for 5 min and washed with PBS, before being resuspended in culture medium to 10^8^ CFU/ml. After removing the Matrigel with cold PBS, organoids were pipetted up and down and were resuspended in culture medium for 1 h. Subsequently, the organoids were reseeded with Matrigel and cultured with medium containing gentamicin (100 μg/ml, Gbico) for 18 h.

### 2.3 The Location of *L. monocytogenes* in Organoids

After centrifugation at 5000rpm for 5min, the collected bacteria were washed once with 1 ml 0.1M NaHCO_3_, re-suspended in a solution containing 0.2 mg/mL fluoresceine isothiocyanate (FITC) dissolved in 0.1M NaHCO_3_ and incubated in the dark at 37°C for 1h. The FITC labeled bacteria were washed twice with PBS and the concentration was set to obtain 10^8^ CFU/mL. Then the labeled bacteria were used to infect crypts before the following steps, and observed in organoids by using a Leica DMi8 Laser Scanning confocal microscope.

### 2.4 Morphology of Intestine Tissue

To observe the pathological change of the intestine, the intestinal tissue was fixed in 4% paraformaldehyde for 24 h, dehydrated in ethanol (for 1h in 70%, 80%, 90%, and 100%, respectively), xylene for 40 s, and embedded in paraffin wax. The paraffin blocks were cut to a thickness of 5 microns and stained with hematoxylin-eosin (H&E) staining.

### 2.5 ELISA

The production of TNF-α was analyzed with Mouse TNF-α ELISA kits (NeoBioscience, China), and Lipopolysaccharide (LPS) was measured with LPS ELISA kits (NeoBioscience, China) according to the manufacturer’s protocols.

### 2.5 Real-Time Quantitative PCR

Total RNA of tissue and organoid samples was extracted using TRIzol (Ambion, USA), after which reverse transcription PCR was performed. Using the primers (**Table 1**), 1 μL of template cDNA was reacted with a master mix in a final volume of 10 μL. The thermal cycling procedure was 30 s at 95°C, followed by 40 cycles of 10 s at 95°C and 30 s at 60°C using an Applied Biosystems 7500 real-time PCR system.

**Table 1:** Primer sequences used for RT-qPCR.

Target genes	Primer sense (5’-3’)	Primer antisense (5’-3’)
*Jag1*	AGTGGCTTGGGTCTGTTGCTTGGT	CATTGTTGGTGGTGTTGTCCTCGGG
*Dll4*	TTCCAGGCAACCTTCTCCGA	ACTGCCGCTATTCTTGTCCC
*Notch1*	CTTGCCAGGTTTTGCTGGAC	CTTTGCCGTTGACAGGGTTG
*Lyz*	GAGACCGAAGCACCGACTATG	CGGTTTTGACATTGTGTTCGC
*Muc2*	ACGATGCCTACACCAAGGTC	TGATCTTTACATGTTCCCA
*GAPDH*	ATGGTGAAGGTCGGTGTGAA	TGGAAGATGGTGATGGGCTT

### 2.6 Immunofluorescence Assay

Intestinal tissue slides were deparaffinized with xylene and rehydrated with an alcohol gradient. To enhance immunoreactivity, the slides were incubated in 10 mM sodium citrate for 15 min at 95°C. Then the slides were cooled to room temperature, washed in PBS for 5 min (five times in total), blocked for 2 h with 5% Bovine Serum Albumin (BSA), and incubated for 2 h with Ulex europaeus agglutinin-1 (UEA-1). Finally, 4′,6-diamidino-2-phenylindole(DAPI)was used to counterstain nuclei. For lysozyme (Lyz) staining, cells were stained with anti-rabbit lysozyme antibody (1:200, Abcam) overnight at 4°C. The samples were incubated with goat anti-rabbit to Alexa Fluor 594 (1:250, Abcam) for 90 min, followed by DAPI for 5 min at room temperature. For *in vitro* imaging, infected organoids were embedded in Matrigel on glass chamber slides. The 0.5% Triton X-100 was used for 20 min to permeabilize the cells. Thereafter, the slides were washed with PBS three times and incubated for 1 h in 5% BSA. Subsequently, UEA-1 and Lyz were used to visualize goblet cells and Paneth cells in organoids, respectively, and the staining was observed with a Leica DMi8 Laser Scanning confocal microscope (Leica, Germany).

### 2.7 Western Blot

Tissue and cell samples were lysed in RIPA buffer containing a protease inhibitor cocktail. Protein concentration in the lysed sample was detected using a bicinchoninic acid (BCA) assay kit (Thermo Scientific, USA). After that, samples containing 5× load buffer were heated for 5 min at 95°C. Equal amounts of protein were separated by 4-20% SDS-PAGE, and transferred to PVDF membranes (BIO-RAD, USA). Then, the membranes were blocked with 5% non-fat milk in TBS with 0.1% Tween-20 for 1 h and incubated with rabbit anti-GAPDH (Abcam, 1:10000), rabbit anti-lysozyme (Abcam,1:1000) overnight, respectively. After the washing, goat anti-rabbit secondary antibodies (Bioss, 1:1500) were used to incubate the membranes. Finally, the optical protein bands were developed using efficient chemiluminescence (ECL) kit, and light emission was captured using the Versa DOC 4000 imaging system.

### 2.8 Statistical Analysis

All statistical analyses were performed using GraphPad Prism 7. A t-test was employed to determine the significant difference between the two groups. The significance levels were shown as *P < 0.05, **P < 0.01 and ***P < 0.001. Data were combined from at least three independent experiments unless otherwise stated and expressed as means ± SD.

## 3 Results

### 3.1 The Intestinal Pathological Changes in Mice After *L. monocytogenes* Infection

**Figure 1:**
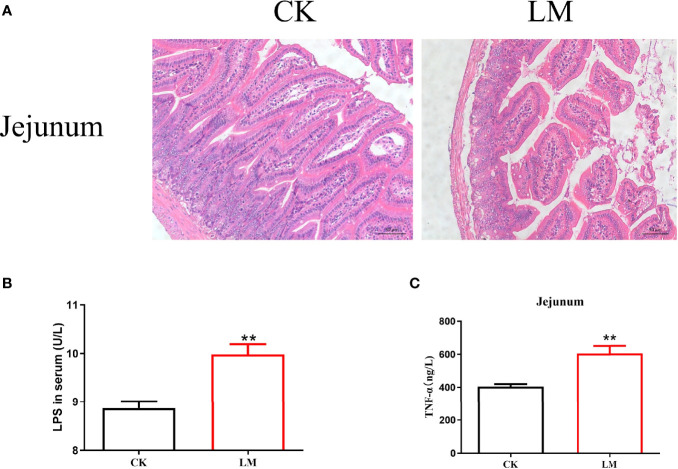
The intestinal pathological changes in mice after *L. monocytogenes* infection. CK, control, orally challenge with PBS; LM, *L. monocytogenes*-infected group. **(A)** Histopathological changes in jejunum tissues were examined by hematoxylin eosin (HE) staining. **(B)** The concentration of LPS was measured in serum. **(C)** The concentration of TNF-α was measured in jejunum. Data is presented as mean ± SD. **P < 0.01. Data combined from at least three independent experiments unless otherwise stated.

Compared with the control group, *L. monocytogenes* infection led to the disorder of intestinal villi in the jejunum (**Figure 1A**). Moreover, after *L. monocytogenes* infection, the concentration of LPS in the serum of mice increased significantly (**Figure 1B**). **Figure 1C** showed that the protein expression level of TNF-α in the jejunum was significantly higher than that in the control group. These results confirmed that *L. monocytogenes* infection could lead to intestinal pathological changes in mice.

### 3.2 The Influence of *L. monocytogenes* on Goblet and Paneth Cells in Mice

**Figure 2:**
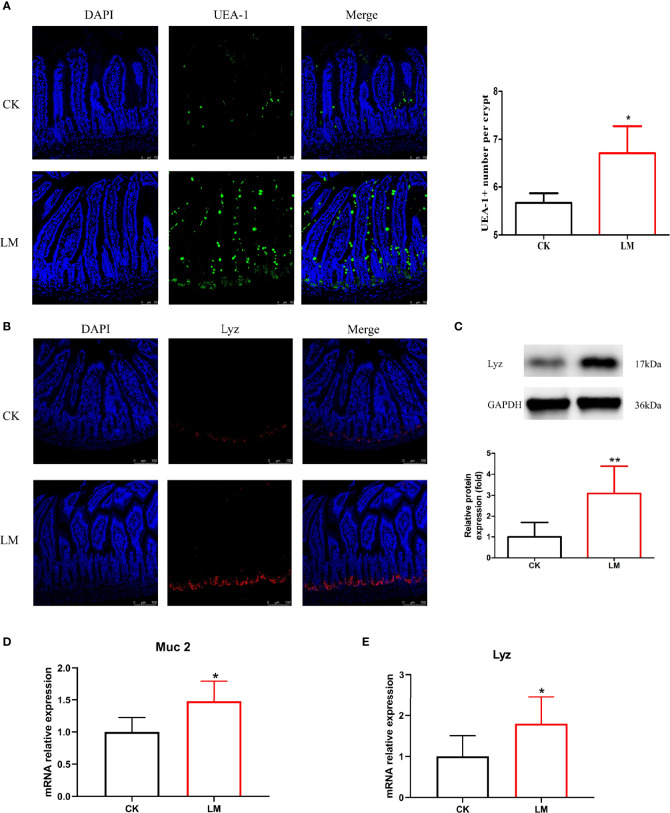
The influence of *L. monocytogenes* on the differentiation of intestinal secretory cells in mice. CK, control, orally challenge with PBS; LM, *L. monocytogenes*-infected group. **(A)** Confocal microscopy analysis of mucus stained with UEA-1 in jejunum sections. **(B)** Confocal microscopy analysis of lysozyme in jejunum sections. **(C)** Western blot of lysozyme in jejunum. **(D)** mRNA levels of *Muc2* from homogenized jejunum samples. **(E)** mRNA levels of *Lyz* from homogenized jejunum samples. Data is presented as mean ± SD. *P < 0.05, **P < 0.01. Data combined from at least three independent experiments unless otherwise stated.

Intestinal secretory cells, such as goblet and Paneth cells, could protect the mucosal barrier and defend against *L. monocytogenes* invasion. The immunofluorescence assay results showed that the oral administration of *L. monocytogenes* could increase the number of goblet cells stained with UEA-1 (**Figure 2A**) and increase the relative expression level of *Muc2* gene significantly in the jejunum of mice (**Figure 2D**). Additionally, the number of Paneth cells stained with *Lyz* was significantly increased through immunofluorescence analysis (**Figure 2B**). The protein and mRNA expression levels of *Lyz* were also upregulated to 3.08-fold and 1.79-fold after *L. monocytogenes* infection, respectively (**Figures 2C, E**). These results indicated that *L. monocytogenes* caused the abnormal increase of goblet and Paneth cells in the jejunum of mice.

### 3.3 The Influence of *L. monocytogenes* on the Notch Signaling Pathway in Mice

**Figure 3:**
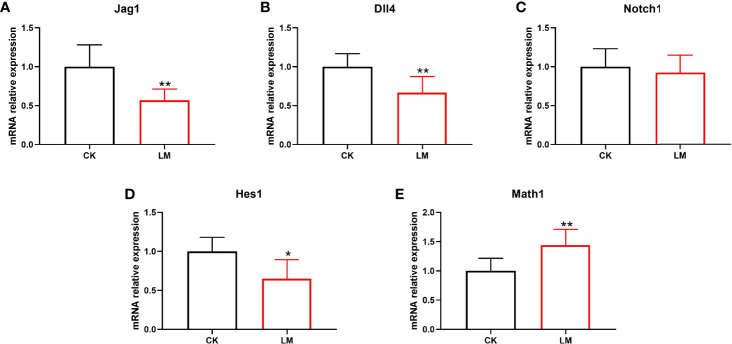
The influence of *L. monocytogenes* on the Notch pathway in mice. CK, control, orally challenged with PBS; LM, *L. monocytogenes*-infected group. **(A-E)** The expression of *Jag1*, *Dll4*, *Notch1*, *Hes1* and *Math1* genes were determined by quantitative RT-PCR and normalized by the expression of *GAPDH*. Data is presented as mean ± SD. *P < 0.05, **P < 0.01. Data combined from at least three independent experiments unless otherwise stated.

The differentiation of intestinal secretory cells was regulated by the Notch signaling pathway, where *Dll4* and *Jag1* are two Notch ligands that regulate the expression of the Notch target gene, *Hes1*. The relative expression of the genes showed that the mRNA relative expression of *Jag1*, *Dll4*, *Notch1*, and *Hes1* were down-regulated to 0.57-fold, 0.66-fold, 0.92-fold, and 0.64-fold, respectively (**Figures 3A-D**). Additionally, the relative expression of *Math1* gene in the jejunum, which governs the differentiation of goblet and Paneth cells, was significantly increased after *L. monocytogenes* infection (**Figure 3E**). These results indicated that *L. monocytogenes* infection could inhibit the Notch signaling pathway, which may be the reason for the increase in the number of goblet and Paneth cells in mice.

### 3.4 The Influence of *L. monocytogenes* on Intestinal Secretory Cell Differentiation and Notch Signaling Pathway in Organoids

**Figure 4:**
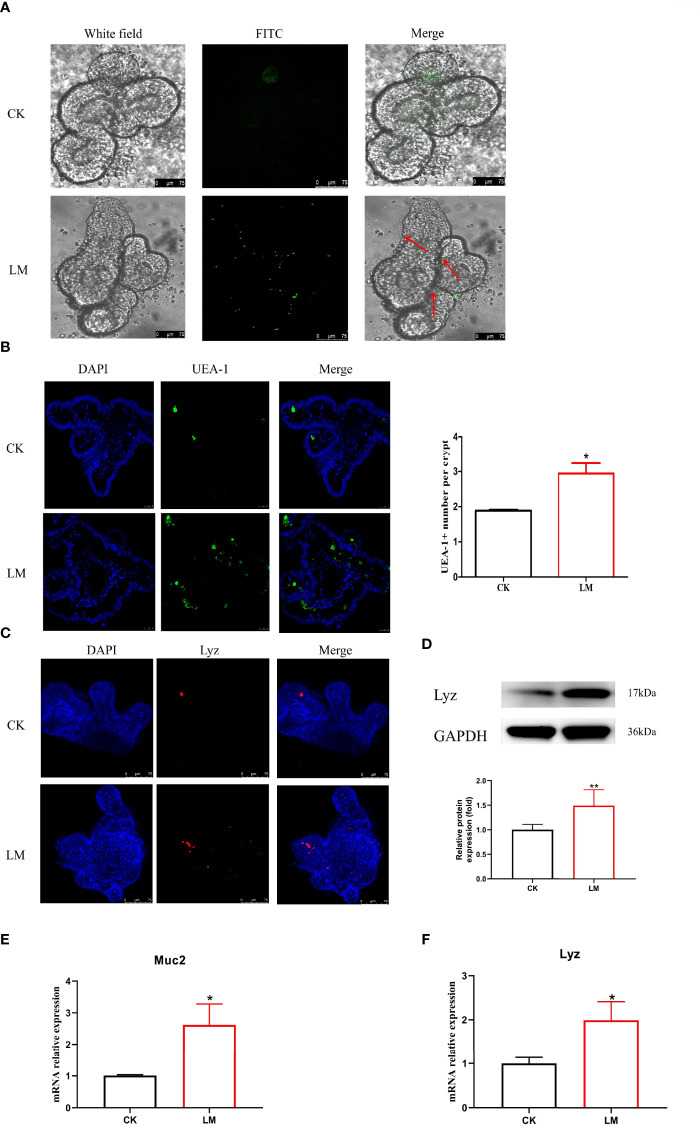
The influence of L. monocytogenes on the differentiation of intestinal secretory cells. CK, control; LM, L. monocytogenes-infected group. **(A)** The location of *L. monocytogenes* in organoids. **(B)** Confocal microscopy analysis of UEA-1+ cells in organoids. **(C)** Confocal microscopy analysis of Lyz+ cells in organoids. **(D)** Western blot of lysozyme in organoids. **(E)** mRNA levels of Muc2 from organoids samples. **(F)** mRNA levels of *Lyz* from organoids samples. Data is presented as mean ± SD. *P < 0.05, **P < 0.01. Data combined from at least three independent experiments unless otherwise stated.

**Figure 5:**
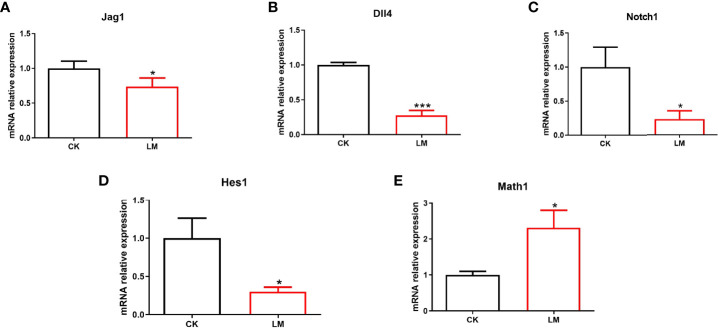
The influence of *L. monocytogenes* on the Notch pathway in organoids. CK, control; LM, *L. monocytogenes*-infected group. **(A-E)** The expression of *Jag1*, *Dll4*, *Notch1*, *Hes1* and *Math1* genes were determined by quantitative RT-PCR and normalized by the expression of *GAPDH*. Data is presented as mean ± SD. *P < 0.05, ***P < 0.001. Data combined from at least three independent experiments unless otherwise stated.

Intestinal organoids, as an *in vitro* model, were used to verify the influence of *L. monocytogenes* on intestinal secretory cell differentiation. **Figure 4A** showed that the FITC-labeled *L. monocytogenes* were observed in organoids, which indicated that *L. monocytogenes* could invade in organoids after 18h co-culture. In addition, results showed that *L. monocytogenes* infection increased the number of UEA-1^+^ cells (**Figure 4B**) and markedly increased *Muc2* expression (**Figure 4E**). Furthermore, the number of Lyz^+^ cells were increased (**Figure 4C**), and the mRNA and protein expression of Lyz were increased significantly after *L. monocytogenes* infection (**Figures 4D, F**), which indicated that *L. monocytogenes* infection increased the Paneth cells of organoids. Also, *L. monocytogenes* significantly decreased the relative expression of *Jag1*, *Dll4*, *Notch1*, and *Hes1* genes and upregulated the relative expression of *Math1* gene in organoids (**Figures 5A-E**). Overall, the results of organoids further confirmed those of mice findings, which also indicated that the inhibition of Notch signaling pathway during *L. monocytogenes* infection may induce the expansion of goblet and Paneth cell population.

## 4 Discussion

*L. monocytogenes* infection is a severe foodborne disease worldwide, reported in more than 30 countries, including the USA, Canada, Germany, Portugal, and Austria (Desai et al., 2019; Jamshidi and Zeinali, 2019). During *L. monocytogenes* infection, the intestine is a vital defense line. This study showed that *L. monocytogenes* infection could lead to villi damage with H&E staining, which was closely related to the increase of pro-inflammatory cytokines TNF-α secretion. This corroborated the study of Alkhuriji et al. that *L. monocytogenes* infection in mice could cause epithelial cells exfoliation and degeneration of the lamina propria and induce the production of TNF-α in the intestine (Alkhuriji et al., 2020). In addition, *L. monocytogenes* increased the level of LPS in the serum, which indicated that the intestinal permeability was increased (Mokkala, K. et al., 2017).

As specialized intestinal epithelial cells, goblet and Paneth cells play an essential role in mucosal homeostasis and bacterial defense. During infection, goblet cell secretion, which contains mucopolysaccharides, critical in forming the first line of defense by protecting the intestinal barrier against pathogenic invasion, was described extensively (Pelaseyed et al., 2014). This study demonstrated that the number of goblet cells, *in vivo* and *in vitro*, and the relative expression of *Muc2* gene were significantly increased, which was consistent with the previous study (Pian et al., 2020). Notably, Paneth cells, located at the bottom of the intestinal crypt, could produce various antibacterial peptides to kill pathogens, including regenerating 3γ (*reg3γ*), lysozyme, and defensin (Bevins and Salzman, 2011). This study showed that the count of Lyz^+^ cells was increased in mice, which was consistent with the protein and mRNA expression levels in mice and organoids. Moreover, it was reported that *L. monocytogenes* increased the number of Paneth cells during co-culture with organoids for 18 h *in vitro* (Huang et al., 2021). Although the increasing number of goblet and Paneth cells shielded against epithelial damage, excessive secretory cells may disrupt the intestinal homeostasis by consuming the stem cells (Putman et al., 2011), which could rejuvenate tissue homeostasis and repair injured tissues (Umar, 2002). Therefore, how to preserve the number and function of goblet and Paneth cells during the treatment of bacterial infection is imperative.

The Notch signaling pathway is a development switch for intestinal secretory and absorptive cells. However, its suppression leads to the inhibition of enterocytes differentiation and a dramatic expansion in goblet and Paneth cell numbers (van Es et al., 2005). The Notch signaling was activated when one of the Delta or Jagged Notch transmembrane receptors interacted with one of the five Notch ligands triggering proteolytic cleavage of the receptor (Scoville et al., 2008). The cleavage released the free Notch 1 intracellular domain (NICD) that translocated into the nucleus to upregulate target genes, mainly of Hes class, such as *Hes1* in the intestine, which suppressed the *Math1* gene (Yang et al., 2001). It was reported that constitutive overexpression of the *Notch1* receptor reduced the differentiation of enteroendocrine and Paneth cells, thus decreasing their numbers (Robine et al., 2005). Also, deficiency in *Hes1* mice led to an abundance of goblet, enteroendocrine, and Paneth cells, but a reduced number of enterocytes (Suzuki et al., 2005). Conversely, the intestine from *Math1* deficient mice exhibited an intestinal epithelium formed only by enterocytes (Yang et al., 2001).

Furthermore, under pathological conditions, Wu et al. found that *Salmonella* infection induced the loss of goblet cells and reduced the mRNA expression of *Muc2* by increasing the expression of *Dll1*, *Dll4*, and *Hes1* genes, indicating the activation of Notch signaling pathway (Wu et al., 2018). However, to the best of our knowledge, the influence of *L. monocytogenes* infection on the Notch signaling pathway has not been documented in mice and organoids. Based on the increase in goblet and Paneth cells, our findings speculated whether *L. monocytogenes* promoted the differentiation of intestinal secretory cells by inhibiting the Notch signaling pathway. Consistent with this hypothesis, the relative expression of related genes of Notch pathway (*Jag1*, *Dll4*, *Nocth1*, and *Hes1*) were detected and decreased, while the mRNA relative expression of *Math1* was upregulated in mice. Also, the intestinal organoids, an effective intestinal cell model, were used to establish an infection model *in vitro*. The mRNA relative expression of *Jag1*, *Dll4*, *Nocth1*, and *Hes1* were down-regulated significantly, while the relative expression of *Math1* gene was significantly increased, which was consistent with a previous study (Huang et al., 2021). As such, the decreased expression of the Notch signaling pathway genes demonstrated *L. monocytogenes* inhibition on the Notch pathway. Combined with these results, the Notch pathway inhibition may be the reason for the increased number of goblet and Paneth cells after *L. monocytogenes* infection in mice and organoids.

In summary, this study indicated that *L. monocytogenes* infection damaged the intestinal barrier and upregulated LPS level in serum, and increased the expression of TNF-α in the jejunum. Furthermore, *L. monocytogenes* infection inhibited the Notch pathway, which may lead to the increasing number of goblet and Paneth cells in the intestine of mice. Therefore, this study provides insight to study the mechanism of *L. monocytogenes* damage to the intestinal mucosal barrier, which is helpful to control *L. monocytogenes* pathogenic infection.

## Data Availability Statement

The raw data supporting the conclusions of this article will be made available by the authors, without undue reservation.

## Ethics Statement

The animal study was reviewed and approved by Nanjing Agriculture University Committee on Animal Resources Committee and the National Institutes of Health guidelines for the performance of animal experiments.

## Author Contributions

CZ: Data curation, Formal analysis, Writing - original draft. YYZ: Writing - original draft. AB: Writing - original draft. JH: Writing - original draft. YFZ: Writing - original draft. KY: Conceptualization, Formal analysis, Funding acquisition, Supervision, Writing - original draft, Writing - review and editing. All authors contributed to the article and approved the submitted version.

## Funding

This work was supported by the National Natural Science Foundation of China (32172267) and Program for Student Innovation through Research and Training (202110307047).

## Conflict of Interest

The authors declare that the research was conducted in the absence of any commercial or financial relationships that could be construed as a potential conflict of interest.

## Publisher’s Note

All claims expressed in this article are solely those of the authors and do not necessarily represent those of their affiliated organizations, or those of the publisher, the editors and the reviewers. Any product that may be evaluated in this article, or claim that may be made by its manufacturer, is not guaranteed or endorsed by the publisher.
